# 

**Published:** 1889-11

**Authors:** 


### CONTENTS.

### COMMUNICATIONS.

The Co-Relation of General and Dental Medicine and Surgery...... 525
The Dentist's Right to practice Medicine .......... 536
Clinical Instruction .......... 540
Address by E. G. Betty .......... 544
Care of Children's Teeth .......... 545
Proceedings Seventh District Dental Society, Cincinnati, Sept. 17, 1889. 548

### SELECTIONS.

Bacteriology and Practice .......... 550
The Medical Student of the Future .......... 553
"He that Bloweth not his own Horn, the Same will not be Tooted.".. 557
A Strange Use of Hypnoptism .......... 559
State Registration of Trained Nurses .......... 560
Notes on Surgical Diseases of Children .......... 561
Microscopical Labratory Notes .......... 562
Babies Beginning to Walk .......... 563
Rules for Good Health .......... 566
Early Rising .......... 567
An Extraordinary Advertisement .......... 568
Micro-Organisms of Dental Caries .......... 569
Officers of the American Medical Association .......... 570

### EDITORIAL.

Bibliographical—Aide Memoirs of the Surgeon Dentist .......... 571
The Post-Graduate of Prosthetic Dentistry .......... 573
Personal .......... 575
Dr. Berry's Death .......... 575

